# What Is Your Diagnosis? A Case of Extreme Thrombocytosis in a Dog

**DOI:** 10.1111/vcp.70022

**Published:** 2025-07-17

**Authors:** Stephanie F. Anderson, King Long Hannah Lee, Megan Dietz, Sarah Guess, Laura White, Mara S. Varvil

**Affiliations:** ^1^ Washington State University, College of Veterinary Medicine, Veterinary Clinical Services Pullman Washington USA; ^2^ Washington Animal Disease Diagnostic Laboratory Pullman Washington USA; ^3^ Veterinary Microbiology and Pathology Washington State University Pullman Washington USA

## 1 Case Presentation

An 8‐year‐old female spayed American Pitbull Terrier was presented to the Washington State University Internal Medicine Service for evaluation of progressive lethargy and weight loss of one‐month duration. On physical examination, the patient was pyrexic (103.9°F), had mild bilateral serous nasal discharge, and a grade II/VI left systolic heart murmur. CBC findings (ADVIA 2120) included a moderate, non‐regenerative (reticulocytes 24 000/μL; reference interval [RI] 0–100 000/μL), macrocytic, hypochromic anemia, neutropenia (2156/μL; RI 2800–13 400/μL), lymphopenia (792/μL; RI 900–4000/μL), and basophilia (880/μL; RI 0–100/μL). A severe thrombocytosis (1 610 000/μL; RI 160 000–500 000/μL) with increased MPV (23.7 fL; RI 7.4–13 fL) was present. Serum chemistry findings (Dimension Xpand Plus) included hypocholesterolemia (111 mg/dL; RI 134–359 mg/dL), low BUN (7 mg/dL; RI 9–26 mg/dL), hypoalbuminemia (2.7 g/dL; RI 2.9–3.8 g/dL), hyperglobulinemia (4.9 g/dL; RI 2.3–4.2 g/dL) and hyperkalemia (8.2 mEq/L; RI 3.7–5.3 mEq/L). Other diagnostics included urinalysis (USG 1.017, otherwise unremarkable), thoracic radiographs (unremarkable), abdominal ultrasound (mild cranial mesenteric lymphadenopathy and mottled splenic echotexture), and echocardiogram (heart murmur likely physiological). Subsequent tests included baseline cortisol (3.65 μg/dL; RI 1–6 μg/dL, Immulite 1000), infectious disease SNAP (IDEXX 4Dx for *Anaplasma phagocytophilum* , *Anaplasma platys* , *Ehrlichia canis* , *Ehrlichia ewingii* , *Borrelia burgdorferi* , and *Dirofilaria immitis*—all negative), and hepatic and splenic fine needle aspirate cytology (interpreted as mild and marked megakaryocyte‐predominant extramedullary hematopoiesis, respectively). Bone marrow aspirate cytology (Figures 1 and 2) and core histopathology were pursued.

**FIGURE 1:**
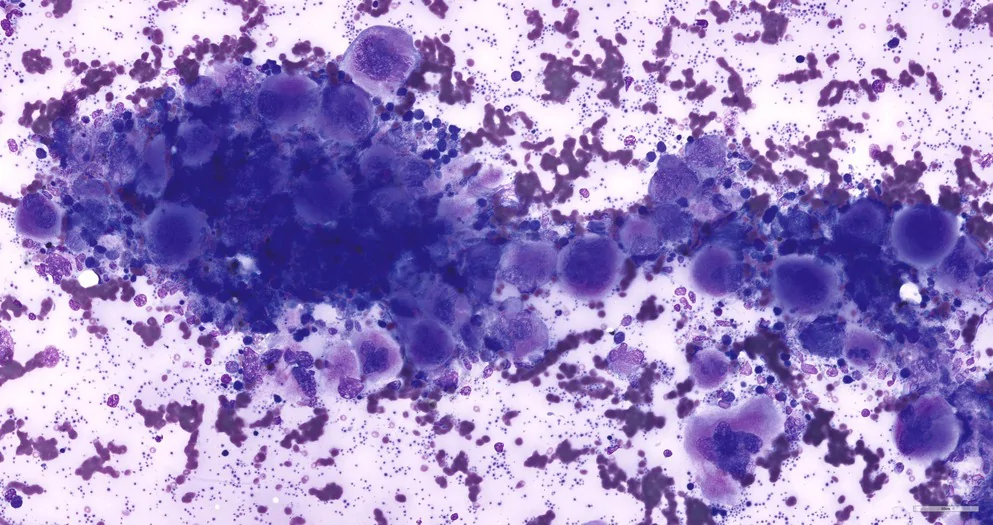
Bone marrow aspirate cytology (×40 objective, Wright‐Giemsa).

**FIGURE 2:**
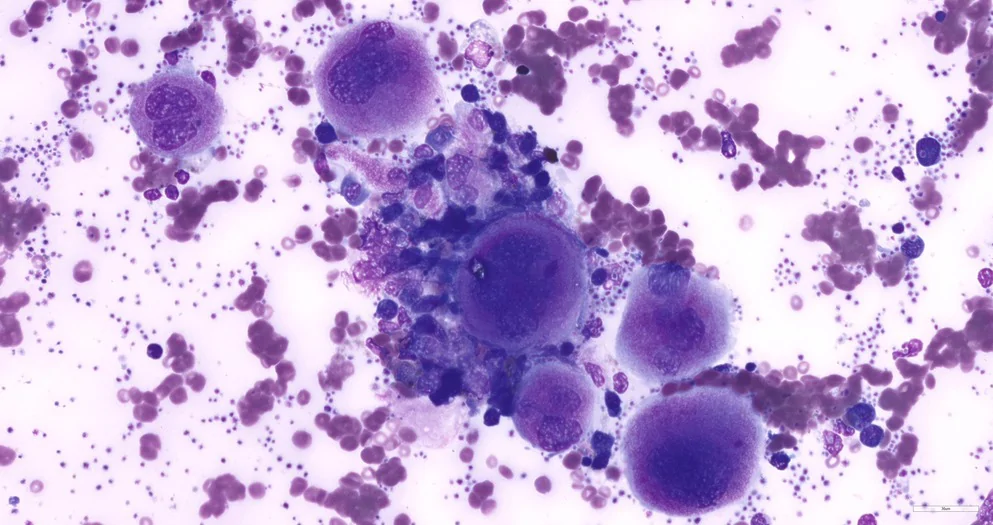
Bone marrow aspirate cytology (×80 objective, Wright‐Giemsa).

## 2 Cytologic Interpretation

Hypercellular bone marrow with markedly increased megakaryopoiesis.

The aspirates contained occasional particles of very densely stained nucleated cells. The particles were markedly hypercellular, with approximately 90% of the particles being composed of nucleated cells. Iron stores were evident within the particles. Megakaryocytes were markedly increased, with approximately 20–30 noted per particle. Morphologic assessment was limited due to the quality of the sample; however, megakaryocytes considered adequate for evaluation consisted of approximately 95% mature cells, characterized by an abundant amount of eosinophilic, granular cytoplasm with hyperlobulated nuclei and coarsely reticular chromatin. Abundant platelets were observed with rare pro‐platelet cylinders. Further cytologic evaluation, including myeloid to erythroid ratio and additional hematopoietic cell morphology, was precluded by the overly dense staining characteristics of the sample.

Bone marrow core biopsies were submitted for histologic evaluation. The bone marrow was hypercellular for age (80%–90%) with trilineage hematopoiesis and < 5% total blasts. The hypercellularity was due to a marked increase and predominance of megakaryocytes with up to 30 megakaryocytes per one 400× high powered field. The megakaryocytes were diffusely and evenly dispersed throughout the marrow and of the predominantly mature type, with corresponding relative increases in megakaryocytes and megakaryoblasts (Figures 3 and 4A). There was frequent hyperlobulation of megakaryocyte nuclei, with up to nine branching bulbous lobes occasionally separated by thin chromatin strands (“staghorn‐like” configuration) (Figure 4B,C). The myeloid to erythroid ratio was approximately 2–2.5:1, attributable to a decrease in erythroid precursors. Both myeloid and erythroid lineages showed maturation to completion. However, both were left‐shifted with decreased numbers of mature cells. Iron storage was adequate on Prussian blue staining. Reticulin staining showed scattered, linear reticulin with minimal to absent intersections (WHO Grade MF‐0) (Figure 4D) [1, 2]. The histologic interpretation was hypercellular marrow with markedly increased megakaryopoiesis and hyperploidy, suggestive of essential thrombocythemia (ET).

**FIGURE 3:**
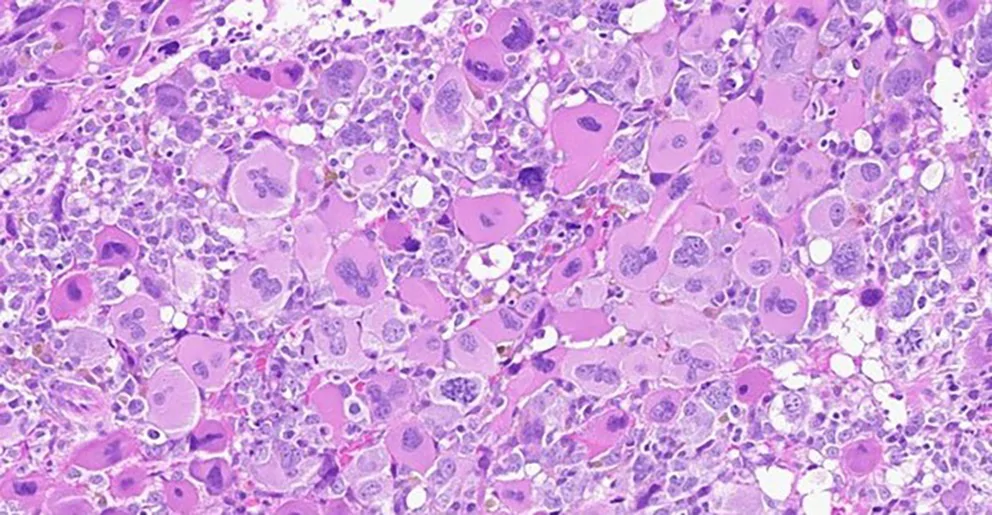
Bone marrow core histopathology (×25 objective, H&E).

**FIGURE 4:**
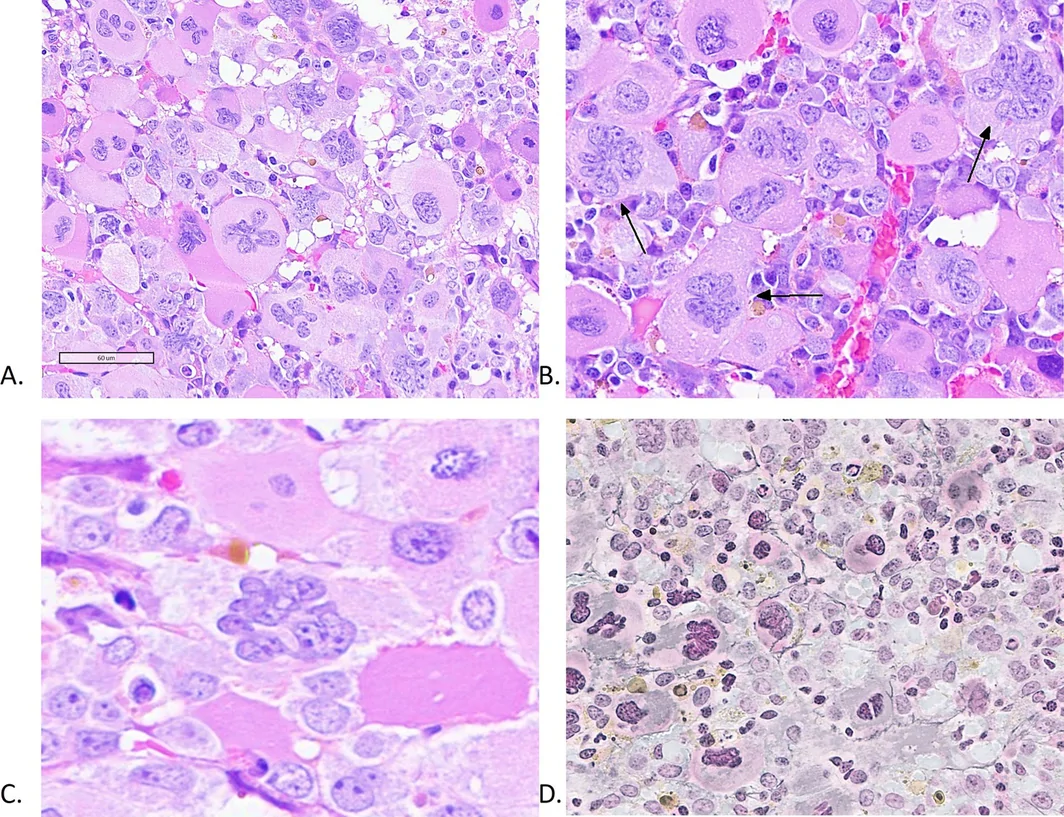
Bone marrow core histopathology. (A) Hypercellular bone marrow composed of sheets of loosely dispersed mature megakaryocytes, 40× objective, H&E. (B) Hyperlobulated megakaryocytes (arrows), ×40 objective, H&E. (C) Staghorn nuclei with bulbous lobules separated by thin strands of chromatin, ×60 objective, H&E. (D) WHO Grade MF‐0 myelofibrosis, ×40 objective, reticulin.

## 3 Discussion

The bone marrow histopathologic findings in conjunction with clinical pathology data are most supportive of ET, an uncommon myeloproliferative neoplasm of megakaryocytes that is rarely reported in humans, canines, and felines [3, 4, 5]. Definitively diagnosing ET is challenging and is mostly considered a diagnosis of exclusion, and is therefore presumptive in this case. Cases of ET are characterized by marked, persistent thrombocytosis (> 450 000 platelets/μL, but often > 1 000 000 platelets/μL) with markedly increased bone marrow megakaryopoiesis of predominantly mature morphology. Frequent concurrent clinicopathologic findings include moderate to marked non‐regenerative anemia, leukocytosis with neutrophilia, +/− basophilia [6]. A noteworthy finding in bone marrow histopathologic sections is the observation of megakaryocytes with “staghorn‐like” configuration characterized by large, mature cells containing deeply lobulated (6–10 lobes), branching or stacked nuclei with bulbous lobules separated by thin strands of chromatin, and abundant mature cytoplasm (Figure 4C). These cells may be observed in cases of Philadelphia chromosome‐negative myeloproliferative neoplasms in people, which include ET, polycythemia vera, and primary myelofibrosis (PMF), and are often indicative of JAK2 mutations [7]. There is considerable clinical and histologic overlap between ET and pre‐fibrotic/early PMF, making the distinction between these conditions challenging. ET is favored in this case due to the predominance of large, mature, hyperlobulated, and staghorn‐like megakaryocytes loosely dispersed throughout the marrow and absence of fibrosis. A pre‐fibrotic PMF cannot be excluded given the overlapping histological features but is less supported due to a lack of tightly clustering or hypolobulated and pyknotic, dysplastic, or bare megakaryocytes commonly observed with this condition [8, 9]. However, sequential bone marrow biopsies to assess progressive fibrosis would be necessary to definitively rule out PMF.

In humans, ET is considered an indolent condition, with patients often presenting asymptomatically or with coagulopathy‐related clinical signs. Clinical signs have been attributed to abnormal platelet function in these individuals, resulting in hypercoagulable platelets that predispose to widespread microvascular thrombosis and a resultant consumptive coagulopathy [5]. To date, this observation has not been demonstrated in veterinary species with presumed ET. In people, an additional diagnostic criterion required by the World Health Organization for a diagnosis of ET is the demonstration of a genetic mutation in either *JAK2*, *CALR*, or *MPL* genes via next‐generation sequencing. A mutation in one or more of these genes has been consistently demonstrated in humans with ET as well as individuals with other myeloproliferative neoplasms [6]. Next‐generation sequencing is not currently widely available in veterinary medicine; however, a *JAK2* mutation has been previously described in a dog with primary polycythemia, another type of myeloproliferative neoplasia [10].

There are several potential causes of thrombocytosis, most of which are attributed to conditions that result in secondary thrombocytosis. Thus, it is necessary to rule out such conditions before considering essential thrombocythemia (ET) as a diagnosis. In dogs, these may include iron deficiency, rebound thrombocytosis following hemorrhage or immune‐mediated disease, chronic inflammatory diseases, paraneoplastic thrombocytosis, endocrinopathies, or indicative medication history [11]. These potential causes were considered unlikely in this case, as adequate stainable iron was observed in the bone marrow sections examined, there had been no clinical or historical evidence of a recent hemorrhagic event, a definitive focus of chronic inflammation was not identified on physical examination or via imaging, the imaging modalities did not identify any discrete masses or concern for other neoplasia, and there was no history of recent medication use in this patient.

Observations that may provide evidence for inflammatory disease in this patient include the documentation of nasal discharge and pyrexia on the physical examination, in addition to the demonstration of hyperglobulinemia on serum chemistry. Although nasal pathology cannot be ruled out in this case, due to the transient and serous nature, the attending clinician considered the nasal discharge to likely reflect mild fluid overload. Pyrexia has been documented in up to 17% of humans with ET [12]. There is currently insufficient data to suggest if the same may be true for canines, thus it is plausible pyrexia could be associated with myeloproliferative neoplasms in dogs. Hyperglobulinemia in this case is unexplained. A suggested hypothesis included an adaptive immune response to the neoplastic megakaryocytes. Serum protein electrophoresis may have assisted in defining this further but was not pursued in this case.

An interesting observation in this patient was the presence of marked neutropenia, in contrast to most reported cases, which tended to be neutrophilic. It is not uncommon for patients with PMF to be neutropenic, often attributed to left‐shifted myelopoiesis, as was observed in this case. Thus, the presence of neutropenia may provide additional conflicting evidence in favor of pre‐fibrotic PMF. Persistent basophilia was noted on the CBC. Peripheral basophilia is not an uncommon feature of myeloproliferative neoplasms and has been associated with the presence of *CALR* gene mutations in humans [13]. The marked hyperkalemia was considered likely pseudohyperkalemia, as is often observed with marked thrombocytosis, attributed to massive release of platelet potassium upon clotting in the serum container.

## 4 Clinical Outcome

The patient was discharged with supportive medications and had follow‐up bloodwork submitted to an external laboratory 1 week later, which showed progressive disease (HCT 20%, platelet count 1 896 000/μL, neutrophils 528/μL, and basophils 2160/μL). The patient was ultimately euthanized approximately 3 weeks later without postmortem examination.

In summary, this is a case of presumed ET, established considering numerous consistent case features, and where other potential causes of thrombocytosis were considered unlikely. A lack of postmortem examination (and therefore ability to definitively exclude other inflammatory or neoplastic conditions), documentation of persistent thrombocytosis over a period of greater than 3 months, and genetic testing for common mutations precluded a definitive diagnosis.

## Conflicts of Interest

The authors declare no conflicts of interest.
